# Role of exosomes in hepatocellular carcinoma and the regulation of traditional Chinese medicine

**DOI:** 10.3389/fphar.2023.1110922

**Published:** 2023-01-17

**Authors:** Man Yao, Shufang Liang, Binbin Cheng

**Affiliations:** ^1^ Oncology Department of Traditional Chinese Medicine, Changhai Hospital, Naval Medical University (The Second Military Medical University), Shanghai, China; ^2^ Faculty of Traditional Chinese Medicine, Naval Medical University (The Second Military Medical University), Shanghai, China

**Keywords:** exosomes, hepatocellular carcinoma, intercellular communication, traditional Chinese medicine, tumor microenvironment

## Abstract

Hepatocellular carcinoma (HCC) usually occurs on the basis of chronic liver inflammatory diseases and cirrhosis. The liver microenvironment plays a vital role in the tumor initiation and progression. Exosomes, which are nanometer-sized membrane vesicles are secreted by a number of cell types. Exosomes carry multiple proteins, DNAs and various forms of RNA, and are mediators of cell-cell communication and regulate the tumor microenvironment. In the recent decade, many studies have demonstrated that exosomes are involved in the communication between HCC cells and the stromal cells, including endothelial cells, macrophages, hepatic stellate cells and the immune cells, and serve as a regulator in the tumor proliferation and metastasis, immune evasion and immunotherapy. In addition, exosomes can also be used for the diagnosis and treatment HCC. They can potentially serve as specific biomarkers for early diagnosis and drug delivery vehicles of HCC. Chinese herbal medicine, which is widely used in the prevention and treatment of HCC in China, may regulate the release of exosomes and exosomes-mediated intercellular communication. In this review, we summarized the latest progresses on the role of the exosomes in the initiation, progression and treatment of HCC and the potential value of Traditional Chinese medicine in exosomes-mediated biological behaviors of HCC.

## Introduction

Hepatocellular carcinoma (HCC) is the most common type of primary liver cancer. HCC usually occurs on the basis of chronic liver diseases, including viral hepatitis, alcoholic and non-alcoholic fatty liver diseases, and cirrhosis. Chronic liver inflammation plays a central role in the hepatocarcinogenesis through multiple mechanisms. Interactions between hepatocytes, hepatic stroma cells, inflammatory and immune cells lead to the production of pro-inflammatory cytokines and chemokines, such as TGF-β, and activation of many key signaling pathways, such as NF-κB and MAPKs pathways. After the tumor developed, cell-to-cell interactions within the tumor microenvironment (TME) induce an inhibitory immune microenvironment and promote the cancer progression and metastasis.

Exosomes, which are small extracellular vesicles with a diameter of 30 nm–150 nm, carry many active components (such as proteins, lipid, RNA and microRNAs), and play an important role in the cell-to-cell communication in the TME. The characteristics of exosomes are usually based on their size or the expression of surface markers, such as CD9, CD37, CD53, CD63, CD81, CD82, TSG101, Alix and Hsp70, etc that could be used in the identification of exosomes (Huang et al., 2018). Currently, many studies have shown that exosomes can be secreted by different types of cells and released into the TME, promoting the proliferation, angiogenesis, migration, invasion, metastasis, and drug resistance of tumor cells (Hao et al., 2022). In addition, the exosomes are also closely related to the occurrence of HCC (Han et al., 2019). This review mainly focused on the role of exosomes in the tumorigenesis, development, diagnosis and treatment of HCC. We also further expounded the value of exosomes for Traditional Chinese Medicine (TCM) in the treatment of HCC.

## Exosomes in HCC diagnosis and prognosis

Due to the absent of early signs, most HCC patients are diagnosed at the intermediate and late stages. Delayed diagnosis of HCC often deprives patients of the opportunity for radical resection. In addition, the lack of sensitive biomarkers or diagnostic methods for cancer recurrence and metastasis also restrict the execution of local and systemic therapies, such as radiotherapy, chemotherapy and immunotherapy. Hence, predicting tumors at a relative earlier stage may improve therapeutic approaches, treatment outcomes and the prognosis.

At present, the most commonly used classical biomarkers for the diagnosis of HCC are alpha fetoprotein (AFP), AFP-L3 and prothrombin induced by vitamin K absence-II (PIVKA-II) (Wang X et al., 2022). However, the AFP level is also increased under pregnancy, hepatitis, cirrhosis and other benign diseases (Wong et al., 2015). In addition, the serum AFP is always at a low level regardless of disease occurrence and progression in about 35%–40% HCC patients. The serum PIVKA-II level is influenced by many factors, such as abnormal coagulation function, cirrhosis, *etc.*, which can cause its level to rise. In recent years, despite new breakthroughs in the diagnosis and treatment methods, the early diagnosis of HCC is still very difficult, and the 5-year survival rate of HCC is no more than 20%. Therefore, there is a clear need for the development of novel, accurate and less invasive methods for early diagnosis of HCC.

Many studies have shown that exosomes are the ideal biomarkers for the early diagnosis of HCC, prediction of tumor recurrence and therapeutic effects. Exosomes have got widely attentions due to their critical role in regulating genes of tumor cells and altering the cell-to-cell communication pathways. Recently, exosomal non-coding RNAs and other cargo molecules have become of interest as candidate biomarkers of HCC diagnosis, recurrence and prognosis (Table 1) (Xue et al., 2019; Wei et al., 2022). Wang et al. (Wang et al., 2018) screened the differentially expressed exosomal miRNAs between HCC and cirrhosis patients and found the combination of miR-122, miR-148a, and AFP could be applied for distinguishing early HCC from liver cirrhosis. Ghosh et al. (Ghosh et al., 2020) showed the combination of exosomal miR-10b-5p, miR-221-3p and miR-223-3p could be taken as a sensitive diagnostic marker for HCC with low AFP. Yao et al. (Yao et al., 2020) identified differentially expressed serum exosomal lncRNAs lnc-FAM72D-3 and lnc-EPC1-4 in HCC and suggested they may be taken as potential candidate biomarkers for HCC diagnosis. Zuo et al. (Zuo et al., 2022) analyzed the differentially expressed liver exosome-related genes and constructed an exosome-related prognostic model, indicating the higher risk score is associated with higher expression of immune checkpoint molecules, including programmed death ligand 1 (PD-L1), programmed death ligand 2 (PD-L2), T-cell Ig and ITIM domain (TIGIT), and indoleamine-2,3-dioxygenase 1 (IDO1).

**TABLE 1:** Potential exosomal biomarkers for HCC diagnosis, recurrence and prognosis.

Candidate exosomal biomarkers	Biofluid	Potential mechanisms	Potential clinical significance for HCC	Ref
miR-122, miR-125b, miR-145, miR-192, miR-194, miR-29a, miR-17-5p and miR-106a↑	Serum		Biomarkers for diagnosis	Xue et al. (2019)
miR-106a↑	Serum	Promoting cancer cell proliferation and invasion	Biomarker for poor prognosis	Xue et al. (2019)
miR-370-3p↓, miR -196a-5p↑	Serum	Promoting cancer cell proliferation, invasion and migration	Biomarkers for diagnosis and poor prognosis	Wei et al. (2022)
combination of miR-122, miR-148a↑, and AFP	Serum		Distinguishing early HCC from liver cirrhosis	Wang et al. (2018)
combination of miR-10b-5p, miR-221-3p and miR-223-3p↑	Serum		Distinguishing low AFP-HCC	Ghosh et al. (2020)
lncRNAs lnc-FAM72D-3 ↑, lnc-EPC1-4↓	Serum	lnc-FAM72D-3 functions as an oncogene, lnc-EPC1-4 functions as a tumor suppressor gene	Biomarkers for diagnosis	Yao et al. (2020)
PD-L1, PD-L2, TIGIT, IDO1	exoRBase		Biomarkers for prognosis	Zuo et al. (2022)
miR-18a, miR-27a and miR-20b↑	Plasma		Biomarkers for metastasis and prognosis	Huang et al. (2021)
miR-125b↓	Serum	Inhibiting migration and invasion and the mRNA expressions of MMP-2, MMP-9, and MMP-14	Biomarker for diagnosis and metastasis	Kim et al. (2021)
exosomal DNA TP53 mutation↑	Serum		Poor RFS and prognosis	Li Y et al. (2022)
combination of miR-29a, miR-29c, miR-133a, miR-143, miR-145, miR-192, and miR-505↑	Serum		Biomarkers for early diagnosis	Lin et al. (2015)
miR-21-5p and miR-144-3p ↑	Serum and HCC tissues		Biomarkers for early diagnosis	Pu et al. (2018)
miR-21-5p↑, miR-92a-3p↓	Serum		Biomarkers for diagnosis	Sorop et al. (2020)
miR-34a↓	Serum		Biomarker for diagnosis and poor prognosis	Chen S. L et al. (2022)
miR-4661-5p↑	Serum		Biomarker for diagnosis	Cho et al. (2020a)
miR-10b-5p↑	Serum and HCC cell lines		Biomarker for early-stage HCC	Cho et al. (2020b)
miR-215-5p↑	Serum and HCC cell lines		Associated with poor DFS disease-free survival	Cho et al. (2020b)
miR-483-5p↑	HCC tissues and plasma	Promoting HCC cell proliferation by targeting CDK15	Biomarker for diagnosis	Lin J et al. (2022)
combination of miR-101 and miR-125b↓	HCC tissues and serum		Biomarker for diagnosis	Sun et al. (2021)
RNA-224↑	Serum	Promoting HCC cell proliferation and invasion	Biomarker for diagnosis and lower OS	Cui et al. (2019)
miR665↑	HCC tissues and serum	Promoting HCC cell proliferation by targeting MAPK/ERK	Biomarker for diagnosis and lower OS	Qu et al. (2017)
miR-18a, miR-221, miR-222, miR-224↑; miR-101, miR-106b, miR-122, miR-195↓	Serum		Biomarkers for diagnosis	Sohn et al. (2015)
miR-122 and miR-148a↓	Serum	Targeting PAX2	Biomarkers for diagnosis and associated with prognosis	Deng et al. (2022)
miR-21 and lncRNA-ATB↑	Serum		Predictors of mortality and disease progression and correlated with poor OS and PFS	Lee H et al. (2019)
miR-92b↑	Serum	Suppressing CD69 on NK cells and promoting cancer cell migration	Predictor of recurrence post liver transplantation	Nakano et al. (2019)
miR-320d↓	Serum		Biomarker for diagnosis and correlated with lower OS and DFS	Li F et al. (2020)
miR-638↓	Serum	Decreasing Ve-Cadherin and Zo-1 of Endothelial Cells	Biomarker for poor OS	Shi et al. (2018); Yokota et al. (2021)
miR-125b↓	Serum		Biomarker for recurrence and reduced TTR and OS	Liu et al. (2017)
hnRNPH1 mRNA↑	Serum		Discriminating HCC from CHB and predicting worse OS	Xu et al. (2018a)
ENSG00000258332.1, LINC00635↑	Serum		Identifying HCC from CHB and predicting worse OS	Xu et al. (2018b)
lncRNA-HEIH↑	Serum		Identifying HCV-related HCC from CHC	Zhang Z et al. (2017)
lncRNA-RP11-513I15.6, miR-1262, and RAB11A↑	Serum		Discriminating HCC from CHB and healthy control	Abd El Gwad et al. (2018)
lncRNA CRNDE↑	Serum		Biomarker for diagnosis and poor prognosis	Wang Y et al. (2021); Huang and Zhang. (2022)
circ0028861↓	Serum		Biomarker for diagnosis of HBV-derived HCC	Wang T et al. (2021)
circ0051443↓	Serum		Biomarker for diagnosis	Chen et al. (2020)
circ0070396↑	Serum		Distinguishing HCC from CHB and liver cirrhosis	Lyu et al. (2021)
circAKT3↑	Serum		Predictor of recurrence post-surgery	Luo et al. (2020)
KV311↓, LBP↑	Serum		Discriminating HCC from CHB	Ye et al. (2022)
miR-10b-3p↓	Serum	Overexpression of miR-10b-3p enhancing sorafenib-induced apoptosis by targeting cyclin E1	sorafenib resistance	Shao et al. (2022)
miR-30d↑	Serum and HCC cell lines		Sorafenib effectiveness	Kohno et al. (2020)
miR-200c-3p, miR-222-5p, and miR-512-3p↑	Serum	miR-200c-3p decreasing cell migration and invasion; miR-222-5p and miR-512-3p regulating metabolism and exerting pro-tumoral properties	Sorafenib effectiveness	de la Cruz-Ojeda et al. (2022)

↑: Upregulation; ↓: Downregulation; CHB: chronic hepatitis B; CHC: chronic hepatitis C; TTR: time to recurrence; OS: overall survival; DFS: disease-free survival; PFS: progression-free survival

Interestingly, the plasma exosomal miRNAs could also serve as potential metastasis-related biomarkers for HCC (Huang et al., 2021; Kim et al., 2021). Huang et al. (Huang et al., 2021) showed a total of 32 differentially expressed miRNAs in the plasma exosomes of patients with metastatic HCC compared with those without metastasis and the combination of three miRNAs (miR-18a, miR-27a and miR-20b) could discriminate metastatic HCC from non-metastatic HCC. Exosomal miR-125b exerts an anti-metastatic effect through interfering TGF-β1-induced epithelial-mesenchymal transition (EMT) and therefore drastically downregulated miR-125b predicts early metastasis of HCC (Kim et al., 2021). Circulating exosomal miR-1307-5p promotes metastasis and helps predict metastasis in HCC through targeting the tumor suppressor genes SEC14L2 and ENG to promote EMT (Eun et al., 2020). In addition, the exosomes may also predict the risk of HCC recurrence. Circulating exosomal circAKT3 is positively correlated with the recurrence rates and higher mortality in HCC patients undergoing surgical treatment (Luo et al., 2020). The circulating exosomal miR-92b may downregulate CD69 and NK cell-mediated cytotoxicity and predicts the risk of posttransplant HCC recurrence (Nakano et al., 2019).

Tyrosine kinase inhibitors (TKIs), including sorafenib, lenvatinib and donafenib are still the first line drugs for advanced HCC, especially for those patients that could not benefit from atezolizumab plus bevacizumab treatment (Bruix et al., 2021; Reig et al., 2022). However, there are still no considerable biomarkers to predict the treatment efficacy. It has been shown that low miR-10b-3p level is associated with sorafenib resistance in HCC (Shao et al., 2022). Exosomal miR-30d is highly expressed in the sorafenib effective patients and thereby maybe serve as a predictive biomarker for the efficacy of sorafenib in HCC (Kohno et al., 2020) . MiR-200c-3p, miR-222-5p and miR-512-3p constitute a biomarker signature of sorafenib effectiveness in advanced HCC (de la Cruz-Ojeda et al., 2022). The baseline miR-200c-3p level is correlated with increased survival, whereas increased miR-222-5p and miR-512-3p levels at 1 month after sorafenib treatment are related to poor prognosis.

## Exosomes in cancer development and progression

**FIGURE 1:**
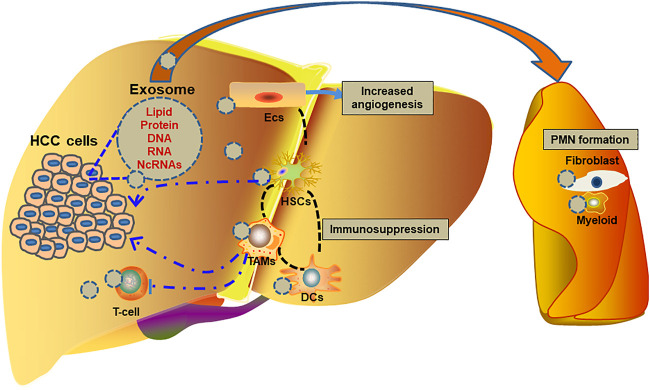
Role of exosomes in the progression of HCC.

Many studies have demonstrated that exosomes are correlated with the development and progression of HCC. In the TME, exosomes transfer bioactive molecules between tumor-tumor cells, tumor-stromal cells and stromal-stromal cells to promote tumor growth, proliferation, and metastasis and create a suitable microenvironment for cancer cells invasion and colonization (Figure 1).

### Tumorigenesis and progression

It has been well recognized that exosomes could represent a contributory factor in the hepatic tumorigenesis, growth and metastasis by regulating the intercellular signal transduction between tumor and normal cells or tumor and tumor cells. Chronic exposure to arsenite contributes to the malignant transformation of hepatocyte. CircRNA 100284, which is involved in the malignant transformation of hepatocyte after arsenite exposure is embodied in the exosomes released by transformed cells and transferred into normal cells (Dai et al., 2018). Exosomal circRNA 100284 accelerates cell cycle and proliferation of normal liver cells by targeting miR-217 to promote the malignant transformation (Dai et al., 2018).

LOXL4, a member of the lysyl oxidase (LOX) family, is commonly upregulated in HCC tissues and predicts a poor prognosis (Li et al., 2019). Overexpression of LOXL4 promotes the migration and invasion of HCC *in vitro*, and intrahepatic and pulmonary metastases *in vivo*. HCC-derived exosomes transfer LOXL4 between HCC cells, and thereby promotes HCC cells invasion and metastasis (Li et al., 2019). In addition, HCC-derived exosomal LOXL4 could also activate endothelial cells and promote angiogenesis (Li et al., 2019). Golgi membrane protein 1 (GOLM1/GP73), a serum marker for HCC, is able to promote the tumorigenesis of HCC (Chen et al., 2015). GOLM1-enriched exosomes promote cell proliferation and migration of HCC through activating the GSK-3β/MMP signaling pathway in recipient cells (Gai et al., 2019). Alpha-enolase (ENO1), a key regulatory enzyme in glycolysis, promotes glucose uptake and lactic acid production by tumor cells (Diaz-Ramos et al., 2012; Capello et al., 2016). ENO1 is also enriched in highly metastatic HCC cells and exosomes derived from these cells. The exosomal ENO1 promotes growth and metastasis of HCC with low metastatic potential by regulating integrin α6β4 and the activation of FAK/Src-p38MAPK pathway, suggesting that ENO1 can be transferred to other HCC cells *via* exosome-mediated crosstalk and further promotes HCC growth and metastasis (Jiang et al., 2020). Angiopoietin-2 (ANGPT2) has been verified as a context-dependent antagonist through destroying vascular stability. HCC-derived exosomes carrying ANGPT2 increase the angiogenesis by a Tie2-independent pathway (Xie Y et al., 2020).

The deregulation of miRNAs plays an important role in human hepatocarcinogenesis. HCC-derived exosomes containing a cluster of differential expressed miRNAs can modulate TAK1 expression and enhance transformed cell growth through shuttling miRNAs to recipient cells (Kogure et al., 2011; Wei et al., 2015). PTEN, an important anti-oncogene, is generally decreased in HCC. Cao et al. (Cao et al., 2019) showed that uptake of exosomal miR-21 derived from HCC cells by other HCC cells could affect cell growth and promote HCC cell proliferation *via* downregulating PTENp1 expression. Liu et al. (Liu et al., 2018) reported that HCC-derived exosomes promoted tumor self-seeding by enhancing the invasive and migration capability of recipient HCC cells through transferring miR-25–5p. Exosomal miR-25 released from cancer cells enhances cell malignant phenotype *in vitro* and tumor growth and metastases *in vivo* through targeting SIK1 (Fu et al., 2022). In acidic conditions, HCC cells release exosomes containing high levels of miR-21 and miR-10b, which promote cancer cell proliferation and metastasis (Tian et al., 2019). In addition, miR-1290 which is overexpressed in HCC patients serum-derived exosomes is able to promote the angiogenesis *via* targeting SMEK1 after delivering into human endothelial cells (Wang S. S et al., 2021).

Circular RNAs (circRNAs) are a type of naturally occurring RNAs which are synthesized by “head to tail” splicing of coding or non-coding RNAs (ncRNAs) (Liu and Chen, 2022). In recent years, the functions of circRNAs on HCC have been deeply investigated. Up to date, more and more circRNAs which could accelerate the progression of HCC have been reported (Wu et al., 2021; Liu Y et al., 2022; Lu Y et al., 2022; Wu J et al., 2022; Shen et al., 2022). The exosomal circCMTM3 is elevated in the serum of HCC patients and HCC cells. CircCMTM3, as a miR-3619–5p sponge, promotes the tumorigenesis of HCC through miR-3619-5p-SOX9 pathway (Hu et al., 2021).The exosomal circRNA-100,338 is highly expressed in metastatic HCC exosomes and promotes the metastasis of HCC *via* enhancing invasiveness and angiogenesis (Huang et al., 2020a). CircFBLIM1 is highly expressed in the serum exosomes of HCC patients and HCC cells, and promotes HCC progression and glycolysis by regulating the miR-338/LRP6 *Axis* (Lai et al., 2020).

Long non-coding RNAs (LncRNAs) have also been demonstrated to play crucial roles in the growth and metastasis of HCC (Wu W et al., 2022). LncRNAs regulate gene expression through sponging miRNA or competing circRNAs. A numbers of lncRNAs have been verified to be elevated in exosomes derived from the tumor cells and/or serum of HCC patients. After transferred into the receipt HCC cells, the exosomal lncRNAs promote the progression of HCC through accelerating the proliferation and metastasis of HCC cells, and suppressing tumor apoptosis (Li B et al., 2018; Wang et al., 2020). Furthermore, exosomal lncRNAs could also promote the angiogenesis of HCC (Li W et al., 2021; You et al., 2021). Insufficient radiofrequency ablation (RFA) may lead to recurrence and metastasis of residual HCC tumors (Su et al., 2018). Ma et al. (Ma et al., 2020) reported that ASMTL-AS1 was upregulated in the tumor after insufficient RFA and could be wrapped by exosomes and then convey malignancy through NLK/YAP axis between cells in residual HCC. In addition, some anti-tumor lncRNAs are decreased in HCC tissues and cell lines. For example, lnc-FAM138B is reduced in HCC tissues and cell lines, and correlated with poor prognosis (Zhuo et al., 2020). Whereas force expressed exosomal lnc-FAM138B may alleviate HCC (Zhuo et al., 2020).

As an important mediator of cell-cell communication, exosomes transport multiple proteins, miRNAs, IncRNAs and circRNAs from HCC cells to normal hepatocytes and tumor cells, and thereby promote the tumorigenesis and progression of HCC through enhancing the proliferation, migration, invasiveness, angiogenesis, et al.

### Formation of the pre-metastatic niche

Extrahepatic metastasis is a main reason for the poor prognosis of HCC and the lungs and bone are the main target organs of HCC metastasis (Zhang et al., 2021; Woo et al., 2022). According to the “seed and soil” theory, the metastatic target organ provides a favorable microenvironment for the extravasation, colonization and growth of the circulating tumor cells, including increase of vascular permeability, local inflammation and immunosuppression (de Groot et al., 2017). Accumulating evidence shows that primary tumors-released soluble factors or exosomes create a favorable microenvironment in the secondary organ for circulating tumor cells arrival and colonization, namely, pre-metastatic niche (PMN) (Peinado et al., 2011; Liu and Cao, 2016; Dong et al., 2021). Tumor-derived exosomes are the key players that mediate the formation of pre-metastatic niche (Morrissey et al., 2021). HCC-derived Nidogen 1-enriched exosomes activate lung fibroblasts and enhance angiogenesis and pulmonary endothelial permeability to facilitate tumor extrahepatic metastasis (Mao et al., 2020). Fang et al. (Fang et al., 2018) showed that high-metastatic HCC cells-derived exosomal miR-1247-3p induced lung fibroblast activation to foster lung metastasis of liver cancer through targeting B4GALT3 and thereby activating β1-integrin-NFκB signaling in fibroblasts. The upregulation of lncRNA HANR in HCC promotes the occurrence of lymphangiogenesis *via* binding to miR-296 and inhibiting the EAG1/VEGF-A pathway in HLECs (Shi et al., 2019). Vascular endothelial cells (ECs) are crucial for vascular remodeling. Huh-7M-derived exosomal miRNAs (miR-638, miR-663a, miR-3648, and miR-4258) may increase the vascular permeability through suppressing the expression of ZO-1 and vascular endothelial-cadherin (VE-cadherin) in ECs and thereby promote intrahepatic tumorigenesis (Yokota et al., 2021). Furthermore, exosomes could also recruit some immune cells into the PMN. Natural killer (NK) cells, the key mediator of the innate immune response, can inhibit tumor initiation and regulate metastatic dissemination. Chen et al. (Chen C et al., 2019) suggested that high-metastatic-potential HCC cells overexpressed miR-561-5p promoted pulmonary metastasis *via* targeting CX3CL1-dependent regulation of CX3CR1+ NK cells infiltration.

### Regulation of epithelial-mesenchymal transition

EMT causes epithelial cells to lose their polarity and adhesion property and acquire the ability to disseminate and invade. EMT plays a crucial role in the cancer cells invasion and metastasis, generation of circulating tumor cells and cancer stem cells, and resistance to chemo- and radiation therapies. Exosomes from highly metastatic MHCC97H cells promotes the migration, chemotaxis and invasion of low metastatic HCC cells by inducing EMT *via* MAPK/ERK signaling (Chen et al., 2018). It has been reported that a variety of miRNAs enriched in the exosomes of HCC cells promote liver cancer EMT and metastasis. Upregulated miR-374a-5p in exosomes of EMT-HCC cells promotes the migration and invasion of Hep3B and 7,721 cells. High-metastatic cancer cells-derived exosomal miR92a-3p promotes EMT and metastasis of low-metastatic cancer cells by regulating PTEN/Akt pathway in HCC (Yang et al., 2020). In the exosomes derived from TGF-β1-treated HCC cells and the serum of HCC patients, the expression of miR-4800-3p expression is upregulated (Lin H et al., 2022). Exosomal miR-4800-3p promotes the EMT and stemness of low-metastatic HCC cells through targeting STK25-mediated Hippo signaling pathway (Lin J et al., 2022). Local hypoxia induces the production of exosomal miR-123f by HCC cells, and miR-123f, in turn, enhances the EMT of HCC cells by targeting LHX6 through inhibiting the Wnt/β-catenin pathway (Yu et al., 2019). Other non-coding RNAs, such as circRNA could also induce EMT in HCC (Liu W et al., 2022). Overexpression of HCC exosomal circ-0004277 enhances the proliferation, migration, and EMT of HCC cells *via* inhibition of ZO-1 (Zhu et al., 2020). In addition, other cargos in HCC-derived exosomes also induce EMT and promote tumorigenesis through multiple signaling pathways, such as Hedgehog pathway (Li L et al., 2021).

## Exosomes in cancer therapy

More than 70% of patients with HCC are diagnosed at mid- or advanced stages and thereby lose the opportunity for radical hepatectomy. Systemic therapies, including chemotherapy, targeted therapy and immunotherapy are still the main methods for advanced HCC. However, the severe adverse events induced by these drugs are a major obstacle for the treatment. Exosomes are widely involved in the transport of biomolecules, including proteins, amino acids, lipids, non-coding RNAs and genetic components to the recipient cells (Tai et al., 2018). Since exosomes have many advantages, such as small size, natural molecular transport properties and good biocompatibility, it could be potentially utilized as a carrier of drugs (Guo and Jiang, 2020; Lin et al., 2020). *In vivo* studies have shown that exosomes can directly deliver mRNA into recipient cells. Furthermore, conventional anticancer drugs may influence the composition and abundance of the cargos. Considering these findings, the exosome-based cancer therapeutics have been extensively explored.

### Exosomes as a delivery vehicle for cancer therapy

Exosomes provide a potential carrier of many types of molecules such as small molecules, biologics, and other therapeutic agents. Chen et al. (Chen J et al., 2021) encapsulated Asiatic acid (AA) into exosomes and investigated the effect of AA-loaded exosomes on HCC cells. AA-loaded exosomes significantly reduced cell vitality, migration, invasion and EMT of HCC compared with free AA, indicating exosomes are potential drug delivery vehicles in HCC treatment (Chen Tet al., 202a). MiR-122 may enhance the chemosensitivity of HCC cells. Adipose tissue-derived MSC (AMSC) exosomes mediated miR-122 transport to HCC cells increases the antitumor efficacy of chemotherapeutic agents and sorafenib on HCC (Lou et al., 2015). Circ-0051443 is lower in the plasma exosomes and tissues of HCC. Exosome-transmitted circ-0051443 induces HCC cells apoptosis and cell cycle arrest and inhibits the xenograft tumors growth *via* the upregulation of BAK1 through miR-331–3p (Chen et al., 2020). Wan et al. (Wan et al., 2022) developed an exosome^RNP^ platform for precise and tissue-specific gene therapies of liver diseases *via* delivery Cas9 ribonucleoprotein complexes. Mo et al. (Mo et al., 2022) constructed exosomes-encapsulated mPEG-PLGA polymer drug-loaded particles and tested the anti-liver cancer effect, targeting ability and biosafety. The data suggest that exosomes could transport the drug to the tumor site with high biosafety and have an effective anti-tumor effect (Scharping et al., 2021). Li et al. (Li X et al., 2022) designed a new targeted delivery exosomes containing a multiplex siRNA (multi-siRNA) capable of simultaneously silencing GPX4 and DHODH. The exosomes with high multi-siRNA loading enhance sorafenib-induced ferroptosis and overcome sorafenib resistance. NK cells-derived exosomes (NK-exo) contain cytotoxic proteins, which may kill various types of tumor cells (Zhu et al., 2017; Di Pace et al., 2020). In HCC subcutaneous and orthotopic animal models, NK-exo significantly inhibits the growth of implanted tumor and exhibits strong tumor-targeting efficacy (Kim et al., 2022). Exosomes, as drug delivery vehicles, have shown huge advantages and encouraging results.

### Role of exosomes in drug resistance

Chemotherapy is a common treatment for malignant tumors. However, HCC is not sensitive to traditional chemotherapy drugs and the effect is not satisfactory. Drug resistance is the main cause of failure of chemotherapy in HCC patients, especially in advanced HCC or metastasis (Tang et al., 2021). Additionally, the exosomes can further reduce the efficacy of chemotherapy for HCC (Giallombardo et al., 2016). Evidences show that exosome-mediated cellular communication is involved in drug resistance (Li Y et al., 2021; Lu et al., 2021; Zhou J et al., 2022). It was reported that circZFR was highly expressed in CAFs and cisplatin-resistant HCC cells (Zhou Y et al., 2022). CAFs-derived exosomes deliver circZFR into HCC, inhibit the STAT3/NF-κB pathway, enhance cisplatin resistance, and promote the tumor progression (Kudo et al., 2018). MiR-27a-3p is upregulated in the exosomes secreted by M2 tumor-associated macrophages (TAMs) and induces cancer stemness and 5-fluorouracil (5-Fu)-resistance of HCC *via* targeting TXNIP (Li et al., 2021). RAB27B, a Rab family small guanosine triphosphate-binding protein, is upregulated in the 5-Fu-resistant Bel-7402 (Bel/5Fu) cells and thereby promotes exosome-mediated drug efflux and causes drug resistance (Hussein et al., 2019). Exosomes from Bel/5-FU cells are also able to deliver miR-32-5p into sensitive Bel-7402 cells, thus inducing angiogenesis, EMT and drug resistance through PI3K/AKT pathway (Fu et al., 2018). In addition, miR-199a-3p that is downregulated in HCC can enhance the chemosensitivity of HCC (Callegari et al., 2018). Exosomes from adipose tissue-derived MSCs (AMSCs) mediated miR-199a-3p delivery to HCC cells sensitizes HCC cells to doxorubicin through mTOR pathway (Lou et al., 2020). Exosomal miR-451a acts as a tumor suppressor and is downregulated in HCC (Zhao et al., 2019). Human umbilical cord mesenchymal stem cells (hucMSCs)-derived exosomal miR-451a suppresses the paclitaxel resistance, cell proliferation, migration and invasion, and promotes apoptosis of HCC cells (Xu et al., 2021).

Sorafenib is the first approved molecular targeted drug for the treatment of unresectable HCC (Llovet et al., 2008). Up to date, sorafenib is also recommended as the first-line treatment for patients with advanced HCC. However, more and more evidences suggest HCC or stroma-cells derived exosomes may induce drug resistance and many patients develop resistance after months of treatment (Tian D et al., 2022). It has been shown that exosomes from HCC cells could induce sorafenib resistance through activating the HGF/c-Met/Akt signaling pathway and inhibiting sorafenib-induced apoptosis (Qu et al., 2016). Hepatitis B core antigen (HBc) increases the expression of exosomal miR-135a-5p which may induce apoptosis protection and chemotherapy resistance in HCC through targeting vesicle-associated membrane protein 2 (Wei et al., 2021). There are also some downregulated miRNAs in HCC tissues and exosomes of sorafenib-resistant liver cancer cells (Wang et al., 2019). Treatment with exosomal miR-744 could reduce sorafenib resistance targeting PAX2 (Wang et al., 2019).

### Role of exosomes in immunotherapy and immune evasion

In recent years, immunotherapy has gained more and more attention, and a variety of drugs, such as pembrolizumab, sintilimab and atezolizumab, have been approved for the clinical application in HCC patients (Finn et al., 2019; Wen et al., 2021; Zhang X et al., 2022). However, the efficacy of immune checkpoint inhibitors (ICIs) in the treatment of HCC is still limited (Rallis et al., 2022). Since HCC develops on the basis of fibrosis/cirrhosis and chronic inflammation, the tumor inflammatory environment is a key player in the initiation, development and metastasis of HCC. Chronic inflammation-induced immunosuppression and immune tolerance are pivotal for the immunotherapeutic resistance of HCC. Exosomes serve important roles in the communication between HCC cells and immune cells, thereby mediating immune evasion and immunotherapeutic resistance (Xie et al., 2018).

Immune cells, including NK cells, dendritic cells (DCs), monocytes, macrophages, myeloid-derived suppressor cells (MDSCs), T-cell, et al., participate in the tumor immunity either by killing tumor cells or by triggering immune evasion (Zhang and Zhang, 2020). In the TME, tumor cells activate various ways to escape the immune system, including inducing immunosuppressive factors, reducing the expression of tumor antigens and promoting immune tolerance (Vinay et al., 2015). The immune cells infiltrated in the TME are the key to mediating antitumor immunity and improving responsive rates of immunotherapy. In HCC, tumor-derived exosomal HMGB1 activates B-cell and promotes T-cell immunoglobulin domain and mucin domain protein (TIM)-1 (+) regulatory B (Breg) cells expansion *via* TLR2/4-MAPK pathway (Ye et al., 2018). TIM-1 (+) Breg cells express high levels of IL-10 and exhibit suppressive activity against CD8^+^ T-cell resulting in HCC immune evasion (Ye et al., 2018). Tan et al. (Tan et al., 2021) and Liu et al. (Liu et al., 2019a) found that HCC-derived exosomes encapsulated and transferred LOXL4 and miR-23a-3p into macrophages, thereby activated the expression of PD-L1 in TAMs to promote apoptosis or inhibit CD8^+^ T-cell function. GOLM1, which is positively correlated with infiltrating TAMs with high PD-L1 expression in HCC tissues, exacerbates CD8^+^ T-cell suppression by transporting PD-L1 into TAMs through exosomes-dependent pathway (Chen J et al., 2021). TIM-3 could inhibit the antitumor immunity after binding with its ligand in a variety of cancers, including HCC. NK cells play a critical role in the innate antitumor immune response. TIM-3 is one of the major inhibitory receptors on NK cells. Overexpressed TIM-3 reduces the antitumor immunity of NK cells and blockade of TIM-3 may be a novel strategy to increase NK function in cancer patients (Ju et al., 2010; Sanchez-Correa et al., 2019). Lu et al. (Lu et al., 2021) found that circTMEM181 was increased in anti-PD1 antibody-resistant HCC patients. Exosomal circTMEM181 sponges miR-488–3p and upregulates CD39 expression in macrophages, thereby induces an immunosuppressive microenvironment and anti-PD1 resistance in HCC (Lu et al., 2021). A higher density of tumoral NK cells is associated with the response to anti-PD1 therapy in tumors (Barry et al., 2018; Lee Y. R et al., 2019). Cancer cell-derived exosomal circUHRF1 induces NK cells exhaustion and causes resistance to anti-PD1 therapy in HCC (Zhang et al., 2020). HCC-derived exosomal circUHRF1 upregulates the expression of TIM-3 in NK cells by degrading miR-449c-5p, and thereby promotes immune evasion and resistance to anti-PD1 immunotherapy in HCC (Zhang et al., 2020). Thus, circUHRF1 might act as a promising therapeutic target in HCC patients. Hang et al. (Huang et al., 2020b) showed that circMET was overexpressed in HCC and promoted HCC development by inducing EMT and immunosuppressive TME *via* the Snail/DPP4/CXCL10 axis.

In contrast, some studies have suggested that exosomes in TME may trigger anti-tumor immunity. In HCC, DCs-derived exosomes containing tumor antigens can stimulate immature T-cell to differentiate into CD8+T-cell and activate their killing ability by increasing the secretion of IFN-γ (Li J et al., 2018b). Rao et al. (Rao et al., 2016) showed that HCC-derived exosomes triggered a DCs-mediated immune response and thereby suppressed ectopic and orthotopic tumor growth in mice. Exosomes derived from AFP-expressing DCs (DEX_AFP_) increase IFN-γ-expressing CD8^+^ T lymphocytes, elevate IFN-γ and IL-2, and downregulate Treg cells, IL-10 and TGF-β in the TME, implying DEX_AFP_ triggers antigen-specific antitumor immune responses in mice with HCC tumors (Lu et al., 2017).

## Exosomes in the TME

Up to date, a lot of papers have identified direct cell-to-cell contacts in the HCC TME, including soluble cytokines, extracellular matrix (ECM) and metabolic products (Colegio et al., 2014; Tripathi et al., 2014; Zhang R et al., 2018; Xu et al., 2019). Increasing evidences indicate that exosomes mediate the complex cross talk between stroma and cancer cells (Wan et al., 2019; Pascut et al., 2020).

### Tumor associated macrophages

In the TEM, the M2 TAMs are the major cell types of macrophages and associate with a poor clinical outcome in HCC. It is well known that M2 TAMs usually promote tumor growth, malignance, and metastasis (Deng et al., 2021; Zhou J et al., 2022). In the TME of HCC, tumor-derived exosomes can promote the M2 polarization of macrophages (Kwon et al., 2020). HCC-derived exosomes could deliver lncRNAs and miRNAs, such as lncRNA TUC339, miR-452-5p and miR-146-5p into macrophages and induce M2 polarization through multiple pathways (Li X et al., 2018; Yin et al., 2019; Zongqiang et al., 2022). In hypoxic condition, HIF-1α increases the number of exosomes carrying HMMR-AS1, which may promote the M2 polarization of macrophages mediated by miR-147a-ARID3A pathway (Wang M. D et al., 2022). HCC cells also modulate the metabolic reprogramming of TAMs through exosomes. HCC-derived exosomal PKM2 induces metabolic reprogramming in monocytes, phosphorylates STAT3 and M2 polarization of macrophages (Hou et al., 2020).

In turn, M2 TAMs-derived exosomes facilitate HCC malignance and eventually accelerate tumor progression and metastasis (Zhang Z et al., 2022). The miR-92a-2-5p in exosomes from macrophages increases liver cancer cells invasion *via* altering the AR/PHLPP/p-AKT/β-catenin signaling (Liu et al., 2020a). Tian et al. (Tian et al., 2021) reported that miR-660-5p-loaded M2 macrophages-derived exosomes augmented HCC development through regulating KLF3. TAMs-derived exosomal lncMMPA facilitates HCC malignance by interacting with miR-548s and increasing ALDH1A3 to promote the glucose metabolism in tumor cells (Xu et al., 2022). M2 macrophage-derived extracellular vesicles facilitate CD8+T-cell exhaustion in HCC *via* the miR-21–5p/YOD1/YAP/β-catenin pathway (Pu et al., 2021). Considering the important role of M2 TAMs in the TME and HCC progression, TAMs have attracted more and more attention for developing new therapeutic strategies for patients with HCC (Cheng et al., 2022).

### Hepatic stellate cells

HCC usually occurs on the basis of chronic liver diseases and advanced fibrosis. As the main source of ECM, hepatic stellate cells (HSCs) play a central role in the hepatic fibrosis/cirrhosis and therefore directly influence HCC formation and progression *via* affecting the TME. It has been shown that activated HSCs infiltrate the stroma of HCC and are associated with tumor progression (Hellerbrand, 2013). Activation of HSC stimulates the release of cytokines, chemokines and growth factors (such as TGF-β, HGF, FGF, EGF, VEGF, et al.), which may aggravate liver inflammation and promote HCC progression (Thompson et al., 2015).

HSCs are also a source of exosomes in the TME. HSCs exosomes-derived circWDR25 regulates ALOX15 expression by sponging miR-4474-3p and ultimately induces EMT in HCC cells (Liu L. H et al., 2022). HSCs exosomes-derived circWDR25 also promotes the expression of CTLA-4 in HSCs and PD-L1 in HCC cells (Liu L. H et al., 2022). Zhang et al. (Zhang W et al., 2022) showed that miR-148a-3p was downregulated in HSCs and HSCs-derived exosomes, and exosomes-depleted miR-148a-3p accelerated HCC progression through ITGA5/PI3K/Akt axis. In addition, HCC-derived exosomes further activate HSCs. Xia et al. (Xia et al., 2021) found smoothened (SMO), the key signal transducer for Hedgehog pathway was low-expressed in quiescent HSCs. HCC cells-derived exosomes transmit SMO to HSCs and activate HSCs through hedgehog pathway. More interestingly, tumor-derived exosome-educated HSCs could also induce drug resistance of hypoxic colorectal tumor cells through regulating lactate metabolism (Li Y et al., 2020).

### Cancer-associated fibroblasts

Cancer-associated fibroblasts (CAFs) are also an important component in the TME of HCC and promote the progression and metastasis (Leonardi et al., 2012). In HCC, CAFs are widely believed to be derived from HSCs (Wang Q et al., 2021). Activated HSCs can be stimulated by cancer cells and then become CAFs (Kubo et al., 2016). Zhou et al. (Zhou et al., 2018) showed that HCC-derived exosomal miR-21 converted HSCs to CAFs by targeting PTEN/PDK1/AKT signaling in HSCs. CAFs promote the malignant biological behavior of HCC by producing various cytokines, non-coding RNAs, ECM and exosomes. Exosomal Gremlin-1 derived from CAFs promotes EMT of hepatoma cells (Qin et al., 2022). CAFs-derived exosomal lncRNA TUG1 promotes liver cancer cells migration, invasion, and glycolysis by regulating the miR-524–5p/SIX1 axis (Lu L et al., 2022). CAFs-derived exosomes also promote the progression of HCC through loss of some tumor suppressors, such as miR-150–3p and miR-320a (Zhang C et al., 2017; Yugawa et al., 2021). Exosomal transfer of anti-tumor miR-29b from CAFs inhibits the migration and invasion of HCC cells (Liu et al., 2020b).

### Endothelial cells

Tumor angiogenesis has been recognized as the leading cause of rapid tumor growth, early metastasis and poor survival. In the TME, endothelial cells (ECs) not only participate in tumor angiogenesis, which is a fundamental process of tumor occurrence, growth and metastasis, also contribute to the immunosuppressive TME through crosstalk with immune cells, such as CD8^+^ T-cell (Sakano et al., 2022). Tumor-derived exosomes can regulate the biological fate of ECs and promote tumor angiogenesis. Huang et al. (Huang et al., 2015) demonstrated that HepG2-derived vasorin could be effectively delivered into human umbilical vein endothelial cells (HUVECs) *via* receptor-dependent endocytosis of exosomes, which is able to promote the migration of HUVECs. SNHG16 and circRNA-100,338, which are upregulated in HCC cells and their secreted exosomes, enhance the proliferative, migratory, and angiogenic abilities of HUVECs after exosomes-mediated transfer from HCC cells (Huang et al., 2020a; Li S et al., 2021). HCC-secreted exosomal miR-210 promotes the tube formation of ECs *in vitro* and enhances angiogenesis by inhibiting the expression of SMAD4 and STAT6 in ECs (Lin et al., 2018). Hypoxia is a common feature of the HCC TME. Exosomes of HCC under hypoxia enhance tube formation by upregulating miR-155 (Matsuura et al., 2019).

Furthermore, exosomes are involved in the production of pro-angiogenic cytokines. Conigliaro et al. (Conigliaro et al., 2015) found that CD90^+^ liver cancer cells-derived exosomes could increase VEGF release and the production of VEGF-R1, hence stimulating angiogenesis through transferring lncRNA H19. In addition, exosomes also regulate angiogenesis-related signaling pathways in HCC. In HCC-derived exosomes, high levels of miR-221 and miR-21 can stimulate the activation of the SAND/NF-κB and STAT3/VEGF signaling pathways, respectively (Liu et al., 2010; Santhekadur et al., 2012).

Anti-angiogenic therapy is a commonly used method in the treatment of liver cancer, such as bevacizumab (D'Alessio et al., 2022). However, the efficacy of anti-angiogenesis is limited by subsequent tumor vasculogenesis and progression (Kuczynski et al., 2016). Zeng et al. (Zeng et al., 2019) reported that the anti-angiogenic therapy triggered VEGF-enriched exosomes release from ECs to promote tumor vasculogenesis. Angiopoietin-2 (ANGPT2), an attractive target for antiangiogenic therapy, induces angiogenesis *via* exosomes derived from HCC cells by a Tie2-independent pathway. Yes-associated protein 1 (YAP1), a core transcriptional regulator of Hippo pathway, is increased in HCC and participates in the tumor angiogenesis (Zhang X et al., 2018). However, YAP1 depletion or inhibition in ECs leads to increased release of exosomes containing the lncRNA MALAT1 into the TME, leading to increased invasion and migration of HCC cells (Li W et al., 2020). These observations suggest exosomes play important roles in remodeling the TME and promoting tumor angiogenesis of HCC.

## Regulation of exosomes by traditional Chinese medicine

TCM has been widely used in the prevention and treatment of HCC and has gained a great deal of attention in recent years (Ling et al., 2018; Gou et al., 2021; Chen S et al., 2022; Chen W et al., 2022). More and more studies showed that TCM formulas, Chinese medicine monomers, or compounds isolated from TCM exert anti-cancer effects by interfering with the TME and influencing the secretion and function of exosomes (Wei et al., 2016; Wu Y et al., 2022). Jianpi Huayu decoction (JHD) has been implicated to be an effective prescription for the treatment of HCC through regulating EMT, immunity, cell cycle and apoptosis (Zhong et al., 2017; Xie J. Y et al., 2020; Liu L et al., 2022). Further study showed that JHD inhibited EMT, migration and invasion of HCC suppressing exosomal miR-23a-3p/Smad signaling (Xie et al., 2022). Jianpi Yangzheng decoction, an effective recipe for advanced gastric cancer, decreases the abundance of serum exosomal PKM2, reduces the delivery of exosomal PKM2 from tumor cells to macrophages, and alleviates exosomal PKM2-induced differentiation of M2-TAMs (Wu W et al., 2022). Dahuang Zhechong Pill, another classical formula, suppresses the metastasis of colorectal cancer through inhibiting exosomal CCL2-induced pre-metastatic niche in liver and M2 polarization of macrophages (Chen E. B et al., 2019). Fei-Liu-Ping ointment downregulates the VEGF, PDGF, IL-6, IL-1β, and TNF-α levels in the serum exosomes of Lewis xenograft model to exert antiangiogenesis roles (Zheng et al., 2020). Coptisine, which is extracted from rhizoma coptidis, blocks the secretion of exosomal circCCT3 from CAFs to reprogram glucose metabolism in HCC (Lv et al., 2020). Shikonin may also reduce the extracellular secretion of exosomal PKM2 and thereby sensitize cisplatin treatment in non-small cell lung cancer (Dai et al., 2022). Astragaloside IV, a major effective component of Astragalus membranaceus, increases the secretion of exosomal miR-126-3p and miR-126-5p from human endothelial progenitor cells and promotes angiogenesis (Xiong et al., 2020). Tanshinone IIA, the main pharmacological component of Radix Salvia miltiorrhiza, protects against chronic obstructive pulmonary disease via exosome-shuttled miR-486-5p (Tian X et al., 2022).

Additionally, exosomes may also be the potential delivery carriers for compounds isolated from TCM (Gutierrez-Millan et al., 2021). Norcantharidin (NCTD), a derivative of Cantharidin isolated from the dried body of Mylabris phalerata Pallas, has multi-target anticancer activities, including HCC (Zhou et al., 2020). Bone mesenchymal stem cell-derived exosomes (BMSC-Exos) carrying NCTD (BMSC-Exos-NCTD) significantly facilitates cellular uptake, inhibits tumor cells proliferation, migration and invasion, and induces cell cycle arrest and apoptosis in HepG2 cells (Liang et al., 2021). In addition, BMSC-Exos-NCTD also suppresses tumor growth *in vivo* than NCTD alone, indicating BMSC-Exos can be used as safe and effective drug-delivery carriers for HCC therapy (Liang et al., 2021). Liu et al. (Liu et al., 2019b) packaged triptolide into exosomes derived from human ovarian cancer SKOV3 cells to construct a triptolide-loaded exosomes (Exos-TP) delivery system. The effects of Exos-TP on proliferation and apoptosis of cells have been confirmed *in vitro* and *in vivo*. Furthermore, Curcumin, a polyphenol compound extracted from Curcuma longa L., has broad-spectrum pharmacological effects, including anti-tumor, anti-inflammatory, and antioxidative activities (Zeng et al., 2018; Shao et al., 2019; Chen Y et al., 2022; Pi et al., 2022). Recently, Abbasifarid et al. (Abbasifarid et al., 2021) reported that HEK-293T cells-exosomes loaded with curcumin eradicate tumor cells through induction of T-cell immune responses in mice. In summary, TCM formulas and compounds isolated from TCM could exert anti-tumor effects through modulating exosomes. Exosomes also play important roles in delivering compounds isolated from TCM to target cells or tissues.

## Conclusion

Up to date, it is still difficult for the early diagnosis and radical treatment of HCC. Exosomes mediate the communication between HCC and non-HCC cells, and thereby act as pro-tumorigenesis factors or tumor suppressors. In this review, we summarized the roles of exosomes in HCC diagnosis, progression, treatment and regulation of TME. Additionally, we also introduced the effect of TCM on the regulation of exosomes in the treatment of HCC. Due to space limitation, many excellent studies are not included in this review. Despite all these achievements, there is still much unknown about the roles of exosomes in HCC. The mechanisms of TCM in the treatment of HCC through regulating exosomes have also not been deeply clarified. Therefore, further investigations are still needed to completely evaluate the clinical application of exosomes in the diagnosis and treatment of HCC and the role of TCM.
